# Tau deposition and structural connectivity demonstrate differential association patterns with neurocognitive tests

**DOI:** 10.1007/s11682-021-00531-7

**Published:** 2021-09-17

**Authors:** Zack Hall, Billy Chien, Yi Zhao, Shannon L. Risacher, Andrew J. Saykin, Yu-Chien Wu, Qiuting Wen

**Affiliations:** 1 Indiana University School of Medicine, Indianapolis, IN, USA; 2 Department of Biostatistics and Health Data Science, Indiana University School of Medicine, Indianapolis, IN, USA; 3 Department of Radiology and Imaging Sciences, Indiana University School of Medicine, 355 West 16th Street, Suite 4100, Indianapolis, IN 46202, USA; 4 Indiana Alzheimer Disease Research Center, Indiana University School of Medicine, Indianapolis, IN, USA; 5 Department of Neurology, Indiana University School of Medicine, Indianapolis, IN, USA; 6 Department of Clinical Psychology, Indiana University School of Medicine, Indianapolis, IN, USA; 7 Stark Neuroscience Research Institute, Indiana University School of Medicine, Indianapolis, IN, USA; 8 Indiana Institute for Biomedical Imaging Sciences, Indiana University School of Medicine, Goodman Hall, 355 West 16th Street, Suite 4100, Indianapolis, IN 46202, USA

**Keywords:** Alzheimer’s Disease, Tau, Diffusion MRI, Structural connectivity, Network metrics, Neurocognitive tests

## Abstract

Tau neurofibrillary tangles have a central role in the pathogenesis of Alzheimer’s Disease (AD). Mounting evidence indicates that the propagation of tau is assisted by brain connectivity with weakened white-matter integrity along the propagation pathways. Recent advances in tau positron emission tomography tracers and diffusion magnetic resonance imaging allow the visualization of tau pathology and white-matter connectivity of the brain in vivo. The current study aims to investigate how tau deposition and structural connectivity are associated with memory function in prodromal AD. In this study, tau accumulation and structural connectivity data from 83 individuals (57 cognitively normal participants and 26 participants with mild cognitive impairment) were associated with neurocognitive test scores. Statistical analyses were performed in 70 cortical/subcortical brain regions to determine: 1. the level of association between tau and network metrics extracted from structural connectivity and 2. the association patterns of brain memory function with tau accumulation and network metrics. The results showed that tau accumulation and network metrics were correlated in early tau deposition regions. Furthermore, tau accumulation was associated with worse performance in almost all neurocognitive tests performance evaluated in the study. In comparison, decreased network connectivity was associated with declines in the delayed memory recall in Craft Stories and Benson Figure Copy. Interaction analysis indicates that tau deposition and dysconnectivity have a synergistic effect on the delayed Benson Figure Recall. Overall, our findings indicate that both tau deposition and structural dysconnectivity are associated with neurocognitive dysfunction. They also suggest that tau-PET may have better sensitivity to neurocognitive performance than diffusion MRI-derived measures of white-matter connectivity.

## Introduction

Tau accumulation is a hallmark pathology of Alzheimer’s disease (AD). Mounting evidence indicates that tau neurofibrillary tangles have a central role in the pathogenesis of AD and are a strong predictor of future brain atrophy and cognitive decline (La Joie et al., 2020). The presence of tau pathology exhibits distinct patterns of anatomical accumulation within neural gray matter. Such patterns have been used to characterize AD staging (Braak & Braak, 1991). This commonly used Braak staging system describes that early on in AD (stage I), tau accumulation begins in the transentorhinal cortex and subsequentially involves the rest of the entorhinal cortex and portions of the anterior hippocampus (stage II) (Braak & Braak, 1991; Braak et al., 2006; Braak et al., 2011). As AD progresses (stages III and IV), tau accumulation spreads into inferior and medial temporal lobes, as well as the posterior cingulum cortex before extending into the neocortex of the frontal and parietal lobes in the final stages (V and VI) of the disease (Braak & Braak, 1991; Braak et al., 2006; Braak et al., 2011). Though originally the staging of tau accumulation in AD was performed using staining methods and immunohistochemistry, with the advancement of neuroimaging techniques it is possible to discern a pattern for the distribution of tau in vivo using positron emission tomography (PET) tracers (Okamura et al., 2018; Villemagne et al., 2015).

The distinct spread pattern and the intracellular origination of tau suggest a key role for white matter (WM) in tau pathology. WM alterations, particularly in axons, in relation to early tauopathy are observed in animal models (Sahara et al., 2014; Nie et al., 2019). In humans, WM degeneration can be studied in vivo using diffusion magnetic resonance imaging (dMRI) techniques (Ji et al., 2019; Stebbins & Murphy, 2009; Wen et al., 2019, 2021). Recent dMRI studies, including our previous work, have revealed tau-related WM degeneration, suggesting that the molecular processes underlying these two phenomena may be interconnected (Kantarci et al., 2017; Strain et al., 2018; Jacobs et al., 2018; Wen et al., 2021). The tau-WM interrelationship is further supported by the observation that AD-related WM degeneration appears to be greatest in fiber tracts that are connected to gray matter regions heavily associated with AD pathology (Lee et al., 2015). Moreover, it has been recently discovered that the spread of tau accumulation is assisted by connective functional brain architecture located within brain WM (Franzmeier et al., 2020; Vogel et al., 2020). Collectively, these findings indicate a tight relationship between tau and WM degeneration, supporting that AD is a dysconnectivity disease (Arendt, 2009; Takahashi et al., 2010).

To better understand how dysconnectivity is implicated in AD pathogenesis, researchers have turned towards the use of graph theory and connectomics (Tijms et al., 2013). Graph theory allows the brain to be viewed as a collection of elements (i.e., nodes, which correspond to gray-matter (GM) regions) and the links (i.e., edges, which correspond to WM connections) which connect the elements (Sporns, 2018; Rubinov & Sporns, 2010). The structural connections of a brain network can be established using dMRI. The use of network metrics derived from graph theory, including clustering coefficients (CC) and node strength, provides insight into the local connectivity of the human brain (Jiang et al., 2017; Masuda et al., 2018; Dennis & Thompson, 2014). Unlike other WM quantitative methods, such as diffusion tensor imaging, that quantify microstructure properties of a specific white-matter tract (i.e. tract-wise changes), the network metrics measure topology and offer the opportunity to examine the network property of a gray-matter ROI (i.e., node-wise connectivity). They provide for quantitative measurements of each node that illustrates how it is connected to the rest of the network. Alterations of brain structural connectivity have been reported in the spectrum of AD. Specifically, significant correlations were found between CC and behavior performances in AD patients (Lo et al., 2010). Accurate classification of AD and prodromal AD has been achieved using network metrics extracted from diffusion MRI (Ebadi et al., 2017; Lella et al., 2018; Lella et al., 2020; Lo et al., 2010; Peraza et al., 2019). Collectively, these studies highlighted the promise of applying structural connectivity to study the dysconnectivity of AD in the preclinical and prodromal phases. Specifically, its relationship with tauopathy is of great research interest, as its correlation with Amyloid-β deposition and brain atrophy have not been consistently reported (Kim et al., 2015; Lombardi et al., 2020; Tucholka et al., 2018; Jacquemont et al., 2017).

Although the improvement of imaging techniques has advanced our understanding of the pathogenesis of AD, clinical diagnosis of the disease still relies heavily on clinical assessments and the use of cognitive examinations (Lane et al., 2018). These examinations cover a wide range of cognitive domains, such as episodic memory, semantic memory, visual memory, and their corresponding immediate/delayed recalls. Each of these tests involves different brain functions which may be associated with different pathophysiological/neurodegenerative processes. Though the two pathological processes – tau and dysconnectivity – appeared to be interrelated, it is unknown whether their effects on various brain functions are disparate or synchronous. Elucidating their impacts on brain function will help connect the dots with regard to multiple pathological processes and shed light on the mechanisms that underlie the cognitive dysfunction seen in AD. The current study aims to address these unknowns by conducting a combined analysis with tau PET and dMRI. This investigates how AD-related tau deposition and structural connectivity are associated with performance on a variety of neurocognitive exams, with a special focus on memory and cognitive function. First, we investigate the correlation between structural network metrics and tau deposition in different brain regions. Secondly, we examine how tau and connectivity independently relate to cognitive performance and whether there exists an interaction effect between the two on cognitive dysfunction.

## Methods

### Participants

Older adults from the Indiana Memory and Aging Study (IMAS) cohort of the Indiana Alzheimer Disease Research Center were recruited who had both tau PET imaging with [18F]Flortaucipir and advanced diffusion MRI data. The participants included cognitively normal individuals (CN) and those with mild cognitive impairment (MCI). MCI subjects were identified based on a multidisciplinary clinical consensus panel review aligning with NIA-AA criteria (Albert et al., 2011). Briefly, MCI participants had significant self-reported or informant/clinician-reported complaints about their cognition, as well as a significant deficit (>1.5 standard deviations below normal) in either memory or another cognitive domain (Albert et al., 2011). Older adults without a measurable cognitive deficit were considered CN participants. Exclusion criteria for neuroimaging were significant cerebrovascular disease or malformations; a history of chemotherapy or radiation therapy; current major depression; a history of schizophrenia, bipolar disorder, developmental disability, Parkinson disease, brain surgery, brain infection, or significant head injury (loss of consciousness >30 min); and/or excessive alcohol consumption. The final cohort included 83 participants: 57 CN and 26 MCI. All participants provided written informed consent according to procedures approved by the Institutional Committee for the Protection of Human Subjects at Indiana University School of Medicine.

### Cognitive assessment

Participants were evaluated using a detailed neuropsychological battery, including measures of memory, attention, executive function, language, visuospatial ability, general intellectual ability, and psychomotor speed. The neurocognitive tests being evaluated in the current study focus on the memory-related tests that are most relevant to AD dementia, including: the Montreal Cognitive Assessment (MoCA) for dementia severity, the Rey Auditory Verbal Learning Test (Rey AVLT) immediate and delayed recall, Craft Story 21 and recall, Benson complex figure copy and recall (Nasreddine et al., 2005). Rey AVLT is designed as a list-learning assessment in which patients are presented lists of nouns and are asked to recall them either immediately or after a 20-min delay (Bean, 2011). The craft stories examination implements a story-based approach to evaluate auditory learning. Patients are presented a detailed story and are asked to repeat details of the story both immediately and after a 20-min delay (Kaur et al., 2018). Benson Figure Recall exam evaluates visuospatial (immediate recall) and memory (delayed recall) ability. During the exam, participants are presented an image of the Benson Figure and are asked to reproduce it immediately and after a time delay (Possin et al., 2011).

### PET

Tau PET scans were performed using a Siemens Biograph mCT scanner. Approximately 10 mCi of [18F]Flortaucipir (18F-AV-1451) was administered intravenously and a 30 min scan was initiated after an uptake time of 75 min. The middle four 5-min frames (80–100 min) were co-registered, averaged, and smoothed with an 8-mm full-width half-maximum Gaussian kernel in Statistical Parametric Mapping 8 (SPM8). The static image volume was then spatially aligned to the subject’s T1-weighted anatomic image. The smoothed images were intensity normalized to the cerebellar crus to create standardized uptake value ratio (SUVR) images. Mean SUVR value was summarized in cortical and subcortical regions-of-interest (ROI) generated by FreeSurfer v6.0 from each subject’s T1-weighted images using the Desikan-Killiany atlas (Desikan et al., 2006). Our analyses included the 70 bilateral FreeSurfer brain ROIs. The thalamus and striatum were not included in the main analyses due to non-specific tracer retention (Lemoine et al., 2018; Lowe et al., 2016; Marquié et al., 2015).

In addition to tau PET, amyloid PET scans were collected using two tracers - [18F]Florbetapir (Amyvid, Eli Lilly and Co., Indianapolis, IN, USA) with a 50-min uptake or [18F] Florbetaben (Neuraceq, Piramal Ltd., Mumbai, India) with a 90-min uptake. Like tau PET, the data were pre-processed using SPM8 and intensity-normalized to the whole cerebellum to create SUVR images. Centiloid value was extracted with the Centiloid algorithm (a form of data normalization that permits grouping data from different amyloid tracers) at the voxel level (Klunk et al., 2015; Risacher et al., 2017). Global amyloid in Centiloid units was extracted using the Centiloid cortical ROI. Centiloid≥20.76 was considered as Aβ positive (Aβ+), as this cutoff best predicted the SUVR cutoffs produced by UC Berkeley (SUVR>1.11 for [18F] florbetapir and SUVR>1.08 for [18F]florbetaben, *data not shown*) (Landau et al., 2013).

### MRI

MRI data were acquired on a single Siemens Prisma 3 T scanner with a 64-channel RF receiver head/neck coil. All participants underwent T1-weighted imaging and multi-shell diffusion MRI. T1-weighted anatomical imaging used a 3-dimensional magnetization rapid gradient echo (MPRAGE) sequence with imaging parameters matching the Alzheimer’s Disease Neuroimaging Initiative 2 protocols (http://adni.loni.usc.edu/methods/documents/mri-protocols/). The diffusion MRI protocol employed a single-shot spin-echo echo-planar imaging (SS-SE-EPI) sequence with a hybrid diffusion imaging (HYDI)-encoding scheme that contained three zero diffusion-weighting (i.e., b-value = 0 s/mm^2^) and five concentric diffusion-weighting shells (b-values = 0, 250, 1000, 2000, 3250, and 5000 s/mm^2^) for a total of 142 diffusion-weighting gradient directions (Wu & Alexander, 2007; 2019, Wen et al. 2019, 2021). The field of view was 240 × 240 mm with an imaging matrix of 120 × 120 and 68 slices with a slice thickness of 2 mm, yielding 2-mm isotropic voxels. An additional b = 0 s/mm^2^ with reversed-phase encoding was acquired for geometric distortion correction.

Diffusion MRI data were first pre-processed using previously described pipelines (Kodiweera et al., 2016; Wu et al., 2018; 2019, Wen et al. 2019, 2021) for noise reduc tion (Manjón et al., 2013), motion and distortion correction (FSL *topup* and *eddy* commands). WM streamline tractography was performed using all 5-shell diffusion data with MRtrix3 guidelines (https://mrtrix.readthedocs.io/en/latest/quantitative_structural_connectivity/ismrm_hcp_tutorial.html (Tournier et al., 2012)). In brief, subject whole-brain streamlines were generated using the multi-shell, multi-tissue constrained spherical deconvolution and probabilistic tracking algorithm (maximum tract length = 250 mm, FA cutoff = 0.06). An anatomically-constrained tractography (ACT) framework with “back-tracking” was implemented for improved reliability of the tractography (Smith et al., 2012). To further boost the robustness and reproducibility of the streamlines, Spherical-deconvolution Informed Filtering (SIFT2) was subsequently applied to filter the reconstructed streamlines and remove the spurious connections (Smith et al., 2015).

All pre-processed PET and MRI images were visually checked by two image analysts (Z.H. and Q.W.). Three datasets were excluded due to motion contamination in the diffusion images.

### Network analysis

For the network edges, two regions were considered structurally connected if there were at least one fiber streamline with two end points that were located in these two regions. Specially, we defined the number of interconnecting streamlines terminated in two regions as the *weights* of the network edges. As a result, we constructed the streamline number weighted structural network for each participant, represented by a symmetric 70 × 70 matrix (70 GM ROIs), the same 70 GM ROIS in tau PET quantification (Supplementary Figure S1 Top left). To characterize the regional network properties (i.e., ROI-wise properties), we focused on two network metrics that are widely used in studying AD-related network changes and are complimentary to one another: clustering coefficient (CC) and Strength (Tijms et al., 2013). These two metrics have also been recently applied to functional connectivity to study the mechanisms through which tau accumulates (Cope et al., 2018; Sintini et al., 2021). CC describes how well neighbors of a given node are connected. It is a measure of *neighborhood* connectivity and can be interpreted as local efficiency (Rubinov & Sporns, 2010). We hypothesize that a lower CC would negatively impact the neurocognitive performances that involve frequent neuronal communication among multiple domains. The CC of node *i* is calculated as: $C_{i}=\frac{2}{k_{i}\left(k_{i}-1\right)}\sum_{j,k\in V}{\left(w_{ij}w_{jk}w_{ik}\right)}^{1/3}$, where *k*_*i*_ is the number of connections between node *i* and its neighboring nodes. *V* is the total number of vertices in the local neighborhood of node *i*. *w*_*ij*_ is the weights (i.e., number of streamlines) between node *i* and its connecting node *j*. Strength quantifies the sum of weights of links connected to the node. Strength provides an indicator of influence of a given *node* to its neighbors, or centrality, and is thus complimentary to the CC. The Strength of node *i* is calculated as the sum of weights of all its connections: $strength_{i}=\sum_{j\in V}w_{ij}$. These two network metrics were extracted using the Brain Connectivity Toolbox (brain-connectivity-toolbox.net) (Rubinov & Sporns, 2010). The robustness of the network metrics has been checked by filtering the connectivity matrix (i.e., streamline density matrix) with a variety of thresholds (Supplementary Figure 1) (Rubinov & Sporns, 2010). The spatial pattern of the network metrics (e.g., Strength) shows high consistency at different thresholds, demonstrating the reliability and reproducibility of the streamline tractography and streamline filtering algorithms. Therefore, we chose to use the original connectivity matrix to reduce the threshold dependency.

### Statistical analysis

For demographic and cognitive variable comparisons, student t-test was employed for continuous variables or χ^2^ tests for categorical variables. Tau deposition and network metrics were pre-adjusted for age, sex, and education in each ROI using linear regression within the whole population. Similarly, neurocognitive tests scores were z-scores after adjusting for age, sex and education. Spearman correlation was performed to evaluate the relationship between two variables, including tau to network metrics, tau to cognitive scores, and network metrics to cognitive scores. We chose Spearman (i.e., non-parametric) over Pearson as some variables do not have normal distribution (e.g. bimodal distribution due to the combination of CN and MCI groups).

To explore whether there is a synergistic effect between tau deposition and connectivity on cognition, we performed the linear regression on cognition with an interaction term between regional tau and connectivity. Age, sex, and education were pre-adjusted for all three variables. Due to the smaller sample size, robust linear regression was applied (rlm function in R) to address the issue that the data may be contaminated with outliers/influential observations.

False discovery rate (FDR) correction was performed among 70 ROIs for all the above analyses. A threshold of *p < 0.05* was considered significant for all statistical models. Statistical analyses were conducted using R-3.6.1.

## Results

### Subject characteristics

Demographics, amyloid-β (Aβ) status, APOE ɛ4 status, and the average test scores are reported in Table 1 for both CN and MCI groups. CN and MCI did not differ significantly with respect to age, sex, or education level. The MCI group was composed of significantly more Aβ positive subjects than the CN group (*p <* 0.01, Table 1). There was no significant difference in APOE ɛ4 status between the two groups. Subjects in the CN and MCI also significantly differed in all tests except the Immediate Benson Figure Recall test. MCI subjects scored significantly lower than their cognitively normal counterparts. Note that cognitive test scores were pre-adjusted for age, sex, and education. All participants have tau PET and diffusion MRI. A few participants are missing Aβ PET due to various reasons, including no-show appointments.

### Tau and network associations

**Fig. 1:**
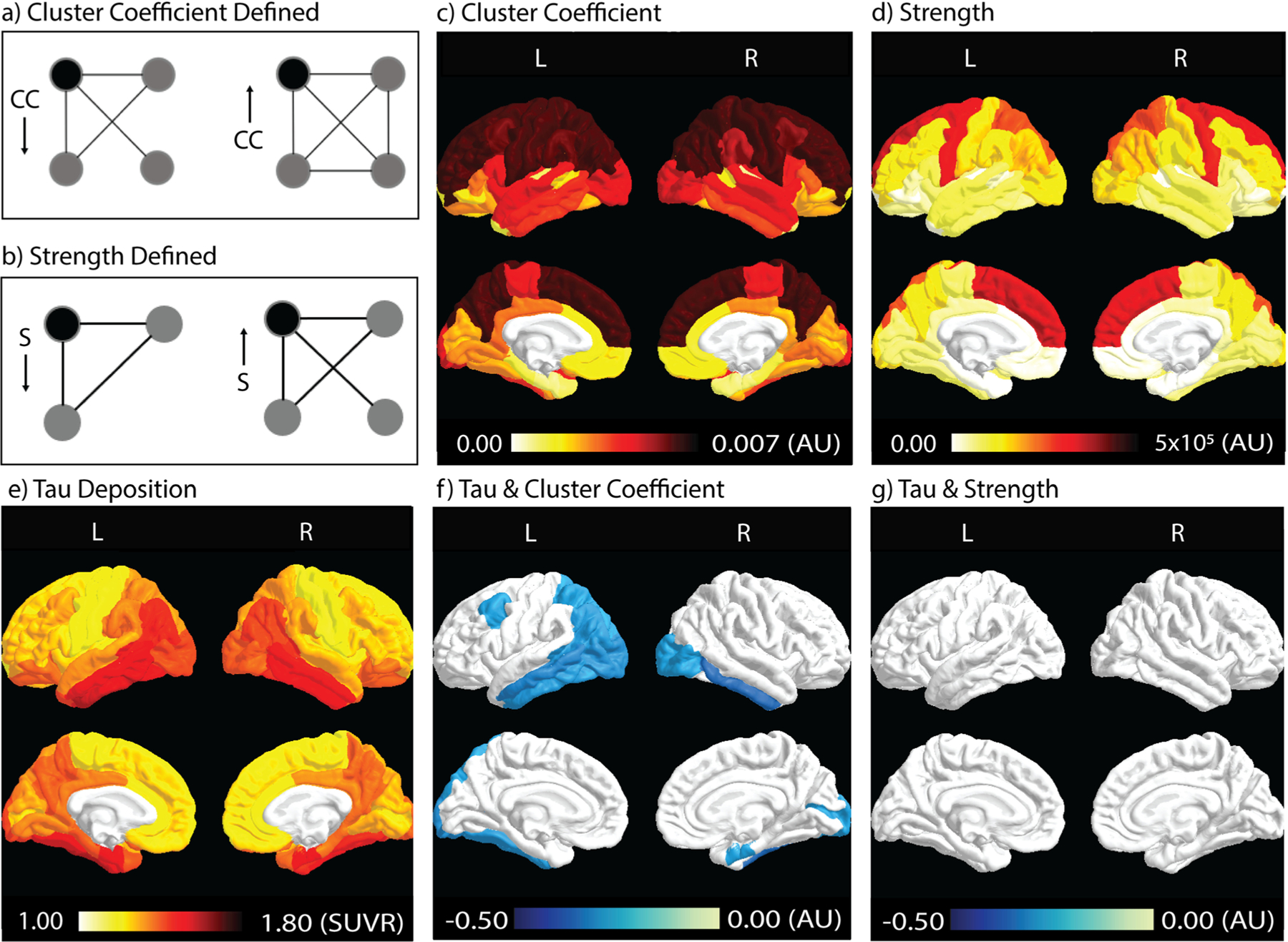
Spatial patterns of tau deposition and WM structural connectivity and their correlations. **a**) CC describes how well neighbors of a given node (black node) are connected. In a binary network (simplified scenario), the clustering coefficient can also be defined in terms of the fraction of triangles in the graph. In this example, CC of the black node on the left is less than that on the right (1/3 vs. 3/3). **b**) Strength measures the number of weighted connections from a ROI (black node) to other regions (gray nodes). The example on the left has a lower strength than the example on the right. CC and Strength describe different entities of a network. In **a**), the two black nodes have the same Strength but different CC. In **b**), the left black node has lower Strength but higher CC. **c**, **d**, **e**) Spatial distribution of CC, Strength, and tau deposition. Dark red indicates higher values in each given measure, while lighter colors indicate lower values. **f**, **g**) Correlation coefficient (Rho) between tau deposition and network metrics. Color-coded ROIs contain negative correlations with a significant level of *p <* 0.05 after FDR correction. Darker blue colors indicate stronger negative correlations (i.e., larger Rho), while lighter blue colors indicate weaker negative correlations (i.e., smaller Rho). CC: cluster coefficient

Spatial patterns of tau deposition and the network metrics are shown in Fig. 1a–e, calculated as the group means of all participants in each ROI. Heuristic definitions of CC and Strength are shown in Fig. 1a and b. CC, a measure of the local efficiency of a gray-matter ROI/node, was highest in the frontal and parietal lobes (in the range of 0.0058–0.0073 arb. unit), while the occipital and temporal lobes had relatively lower CC values (in the range of 0.00057–0.00097 arb. unit) (Fig. 1c). Strength is a measure of influence or centrality of an ROI and was highest in the medial frontal lobe and precentral gyrus (in the range of 2–3 × 10^5^ arb. unit). The parietal lobe showed medium-strength values while all other regions had relatively low strength values (in the range of 1–3 × 10^4^ arb. unit) (Fig. 1d). Tau deposition (Fig. 1e) was highest in the temporal lobe, with parts of parietal and occipital lobes also seeing heavy tau deposition (SUVR in the range of 1.3–1.6).

Correlation coefficients (Rho) depicting the relationship between tau deposition and network metrics are color-coded in ROIs that contain significant correlations (*p <* 0.05, FDR corrected, Fig. 1f, g). Significant correlations between tau deposition and CC (Fig. 1f) are present in the temporal and occipital lobes (*p <* 0.05, FDR corrected). This pattern was similar to the areas of highest tau deposition (Fig. 1e). No significantly correlated areas between tau deposition and Strength were observed after FDR correction (Fig. 1g).

## Associations with neurocognitive performance

### Tau with neurocognitive performance

**Fig. 2:**
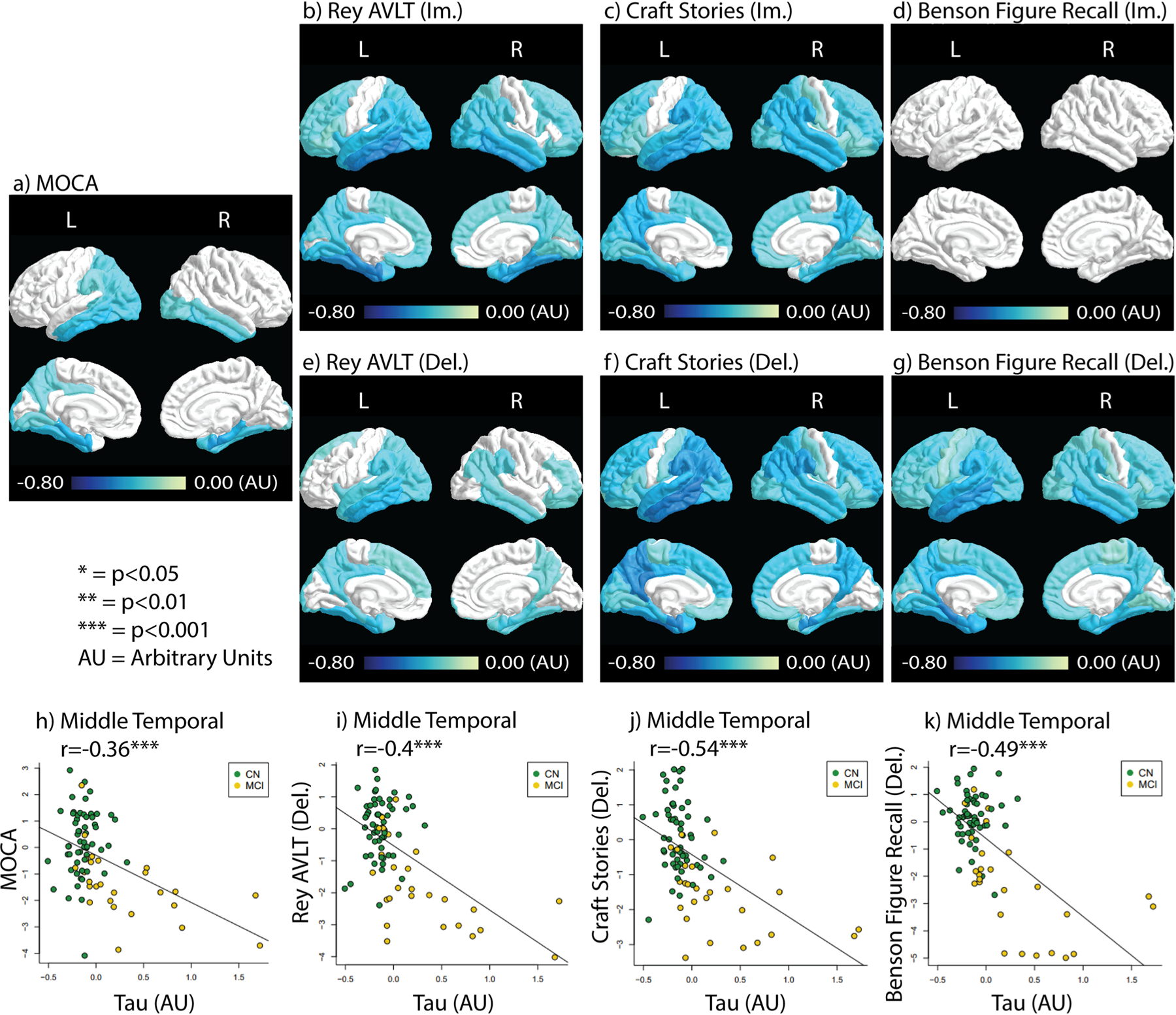
Tau correlation with the neurocognitive test results. Correlation coefficients (Rho) depicting the relationship between tau deposition and test results are color-coded in ROIs that contain significant correlations (*p <* 0.05, FDR corrected). **a**) the MoCA test result correlation with tau. **b**-**d**) Immediate recall test results correlation with tau. **e**-**g**) Delayed recall test results correlations with tau. **h**-**k**) Scatter plots between tau and neurocognitive tests in the middle temporal ROI with color-coding for each study group. The significance level is labeled next to the r-value. ***: *p <* 0.001

Tau correlations with the cognitive tests are shown in Fig. 2. All scores measured except the immediate Benson Figure Recall showed significant negative correlation with tau accumulation across multiple regions of the brain. MoCA and delayed Rey AVLT scores showed relatively fewer ROIs correlated with Tau accumulation (22 and 32 significantly correlated ROIs respectively, Fig. 2a & e). More ROIs correlated with tau accumulation were observed in immediate Rey AVLT (42 ROIs), immediate craft stories (57 ROIs), delayed craft stories (59 ROIs), and delayed Benson Figure Recall (61 ROIs) (Fig. 2b–g). The tests that showed the most strongly correlated ROIs with tau include the immediate Rey AVLT, delayed craft stories, and delayed Benson Figure Recall. Immediate Rey AVLT showed relatively strong negative correlations with tau in the entorhinal cortex (Rho = −0.52) and the inferior temporal lobe (Rho = −0.52). Delayed craft stories showed relatively strong negative correlations with tau in the entorhinal cortex (Rho = −0.51), the inferior temporal lobe (Rho = −0.52), the isthmus cingulate gyrus (Rho = −0.52), and the middle temporal lobe (Rho = −0.53). Delayed Benson Figure Recall scores showed relatively strong negative correlations with tau in the parahippocampal ROI (Rho = −0.52).

Tau did not display any general pattern of being more correlated with the delayed or immediate test scores. Scatter plots between tau deposition and neurocognitive tests in the middle temporal ROI are shown in Fig. 2h–k, with color-coding for each study group. It can be seen that the correlation between tau deposition and test score is mostly driven by the MCI group, likely due to the lack of variance of tau accumulation in CN subjects. Supplementary Figure S2 shows tau accumulation with test score without FDR correction. More ROIs of significance in the non-FDR corrected data are observed as expected. However, Benson Figure Recall (lm) still did not have ROIs with significant correlations.

### Connectivity with neurocognitive performance

**Fig. 3:**
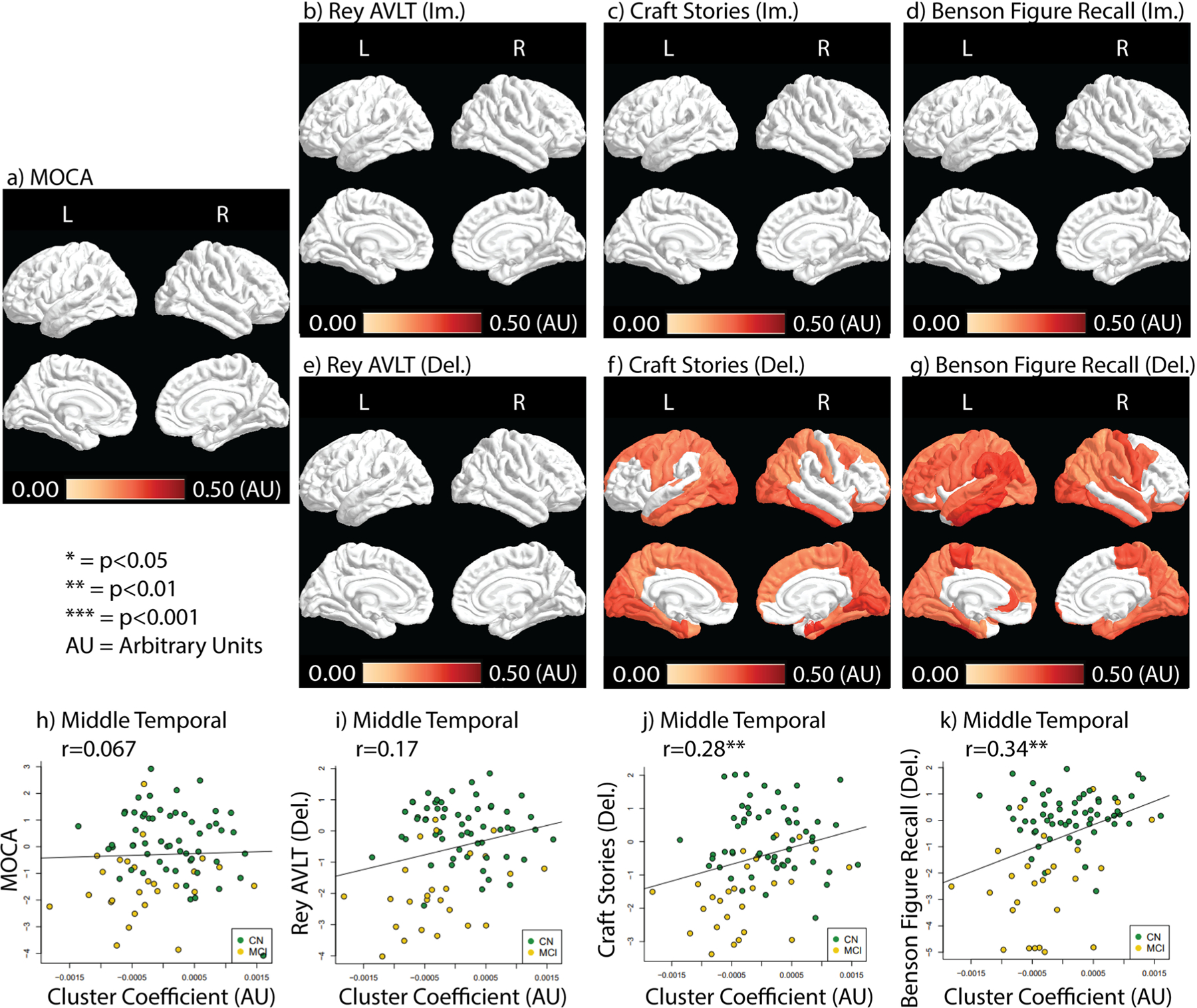
CC correlation with the neurocognitive test results. Correlation coefficients (Rho) depicting the relationship between CC and test results are color-coded in ROIs that contain significant correlations (*p <* 0.05, FDR corrected). **a**) the MoCA test result correlation with CC. **b**-**d**) Immediate recall test result correlation with CC. **e**-**g**) Delayed recall test results correlation with CC. **h**-**k**) Scatter plots between CC and neurocognitive tests in the middle temporal ROI with color-coding for each study group

CC correlations with cognitive tests are shown in Fig. 3. Only delayed craft stories and Benson Figure Recall test scores showed correlations with CC after FDR correction, where higher CC is associated with a higher performance (Fig. 3). Delayed Benson Figure Recall showed more ROIs correlated with CC than delayed craft stories scores (50 vs. 39, respectively) (Fig. 3f and g). Scatter plots between CC and neurocognitive tests in the middle temporal ROI are shown in fig. 3h–k. CC shows positive correlation driven by both CN and MCI groups (Fig. 3h–k). Without FDR corrections, Rey AVLT (lm. & Del.) and craft stories (lm.) showed positive correlations with CC in earlier Braak stage ROIs (Supplementary Figure S3b,c,e).

Supplementary Figure S4 showed Strength correlations with and without FDR correction. Strength demonstrated a different correlation pattern than CC. Without FDR correction, Strength was correlated with at least one ROI in the temporal lobes in all tests except immediate Benson Figure Recall (Supplementary Figure S4a). Rey AVLT and Craft stories (both im. and del.) showed ROIs significantly correlated with Strength outside of the temporal lobe without FDR correction (Supplementary Figure S4b).

With FDR correction, Strength was correlated with test performance in the right inferior temporal ROI for MoCA and immediate Rey AVLT only (r = 0.39, *P* = 0.024 for MoCA and r = 0.44, *P* = 0.0028 for immediate Rey AVLT, FDR corrected) (Supplementary Figure S4a).

### Interaction between tau deposition and connectivity on neurocognitive performance

**Fig. 4:**
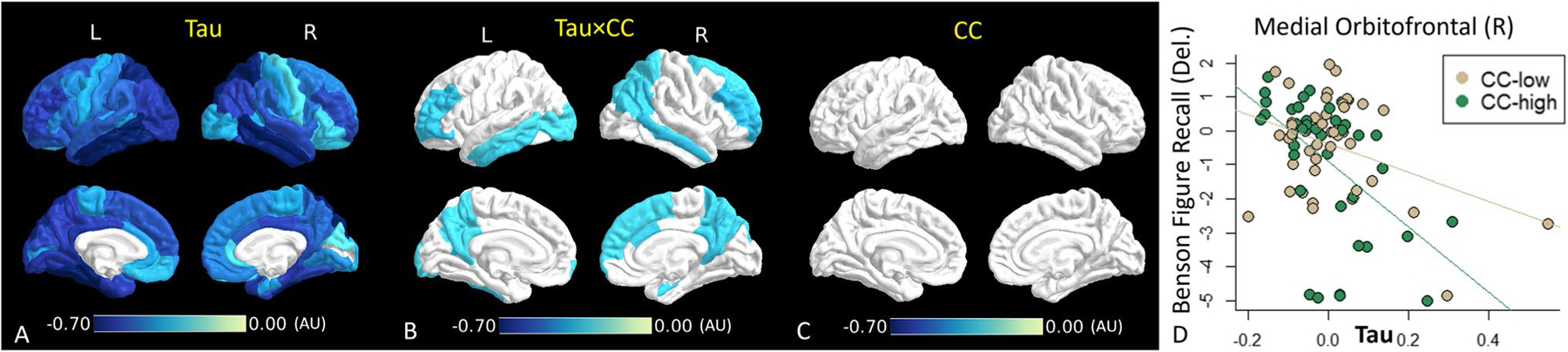
Spatial pattern of interaction between tau and CC on Ben Figure Recall (Del.) using robust linear regression. Color-coded ROIs contain significant correlations (p < 0.05, FDR corrected). Color corresponds to effect size for Tau (**A**), the interaction (**B**), and CC (**C**). **D**. Scatter plot of tau (in Medial Orbitofrontal (Right)) and Benson Figure Recall (Del.) in higher CC (green) and lower CC (brown) groups. AU: arbitrary unit

We performed interaction analyses between tau and CC on neurocognitive tests, including Craft Stories (Del.) and Benson Figure Recall (Del.) where independent associations were found for both tau and CC. After FDR correction, an interaction effect was found for Benson Figure Recall (Del.) (Fig. 4B), implying a synergistic effect between tau and CC on the worse performance of this cognitive test. The direction indicates that a unit increase of tau deposition is associated with more decline in Benson Figure Copy (Del.) in subjects with higher CC than with lower CC (Fig. 4D).

### Effect of Aβ and diagnosis on the association with neurocognitive performance

Scatter plots with color-coding for amyloid status (Aβ+/Aβ-) for the middle temporal ROI are shown in Supplementary Figure S5. Participants with Aβ+ tended to have higher tau and lower cluster coefficient. Associations between global amyloid deposition (global cortex centiloid value) and connectivity metrics were found in selective ROIs including Amygdala, Banks of the superior temporal sulcus, Fusiform, Parahippocampal and Lingual (Supplementary Table 1), which did not survive FDR correction. Non-FDR corrected correlation between tau/CC and test scores split into Aβ+/Aβ-shown in Supplementary Figure S6. Similarly, results for CN/MCI subject groups are shown in Supplementary Figure S7. For both tau and CC, the correlations with cognitive tests were strongest in Aβ+ and in MCI.

## Discussion

By comparing measures of WM connectivity obtained by dMRI (CC and Strength) to tau accumulation obtained by tau PET, the present study shows that tau accumulation is negatively correlated with structural connectivity in the early Braak stage regions. This is consistent with our recent finding that tau-related WM alterations were concentrated in early tau propagation pathways (Wen et al., 2021) and results from postmortem studies that synaptic density loss is related to tau deposition in the spectrum of AD (Pooler et al., 2014; Vanhaute et al., 2020). When compared to neurocognitive tests, tau deposition and network metrics showed disparate patterns. Specifically, tau deposition demonstrated brain-wide associations with all neurocognitive tests except for immediate Benson Figure Recall. At the same time, CC was associated with delayed Craft Stories and delayed Benson Figure Recall and demonstrated a synergistic effect with tau deposition on delayed Benson Figure Recall. These results confirmed that tau PET signal is a highly sensitive biomarker for generalized cognitive decline, whereas CC is more specific to delayed cognitive tasks (Craft Stories and Benson Figure Recall).

The current study focused on the association of the neuropsychological tests and the neuroimaging biomarkers - tau PET and WM connectivity. While Rey AVLT (lm. and Del.), Craft Stories (lm. and Del.), and Benson Figure Recall (Del.) test the memory function, Benson Figure Recall (lm.) evaluates the visuospatial ability (Dodge et al., 2020). Interestingly, the Benson Figure Recall (lm.) is also the only score that did not show group difference between CN and MCI, nor any association with tau or structural connectivity in the present study. In general, AD symptoms begin with a deficit in memory. When the process advances, impairment spreads to other functions, including visuospatial ability (Lindeboom & Weinstein, 2004). Our results echo with this by showing that compared to memory deficit, visuospatial ability played a lessor role in the association with neuroimaging biomarkers compared to memory deficit in the preclinical and prodromal phases. The tight relationship between tau and memory performance is consistent with its central role in the pathogenesis of AD and being a strong predictor of future cognitive decline (Giannakopoulos et al., 2003; Digma et al., 2019; Hanseeuw et al., 2019; La Joie et al., 2020).

On the other hand, WM dysconnectivity only demonstrated associations with delayed memory tasks, including Craft Stories and Benson Figure Recall. The immediate and delayed recall tests are designed to differentiate AD-related dementia from other dementing diseases. Specifically, while the immediate recall tasks can effectively detect general dementing disorders, a significant decline in the delayed recall is more specific to AD-related dementia (Hart et al., 1988; Albert, 1996). This, combined with our findings on WM dysconnectivity, may imply that the network metric extracted using diffusion MRI could be a potential biomarker for AD-related neurodegeneration. Further investigation in a larger cohort is warranted to evaluate these claims.

The interaction analysis further shows a synergistic effect between tau and CC on the delayed Benson Figure Recall exam. Delayed Benson Figure Recall involves memory and visuospatial ability, which requires many brain regions to communicate and work together. According to these findings, the co-occurrence of GM tau pathology and WM dysconnectivity is associated with a worse decline in delayed Benson Figure Recall. In a previous large-scale study, delayed Benson Figure Recall was identified as one of the most sensitive neurocognitive tests at clinically screening for early cognitive impairment (Liew, 2019). Our findings combined with the work of this previous study may suggest that both tau and CC are useful imaging biomarkers for detecting early cognitive impairment.

Additionally, the results elucidated differences between the two measures of network connectivity that were analyzed, CC and Strength. CC was much more heavily associated with performance on the neurocognitive exams than Strength. CC and Strength also demonstrated different association patterns with cognitive tests. Though both are measures of WM interconnectivity, they represent different network properties. CC measures the whole neighborhood connectivity and is closely related to local efficiency (Rubinov & Sporns, 2010). Therefore, it appears reasonable that CC is correlated with complex memory functions such as delayed Benson Figure Recall, which presents a high demand for “efficient” communication and integration of information between different brain regions. Strength measures how tightly a local node is connected to other regions and is more related to a local function. It is more often used to identify the “hubs” regions in a network and has been found less sensitive to AD-related memory deficit than CC (Tijms et al., 2013).

One limitation of the current study is that our main findings are in the combined group of CN and MCI, and combined Aβ status. While this offered a larger sample size that allows multiple comparison corrections to be performed, it did not address the potential difference between these subgroups (e.g., CN vs. MCI, Aβ- vs. Aβ+). We performed group-wise analysis without FDR correction (supplementary material). Similar patterns were observed in MCI and in Aβ+ but not in CN or Aβ-groups. Future work on group-wise analysis based on diagnosis as well as on amyloid status with a larger sample size is warranted.

## Conclusion

Overall, the current study’s findings suggest that the pathophysiological mechanisms behind tau accumulation and WM dysconnectivity are correlated. However, their effects on cognitive function appear to be distinct from one another. While tau deposition demonstrates a more fundamental effect on the overall neurocognitive performance, WM dysconnectivity is associated with delayed memory recall in craft stories and Benson Figure Copy. Tau and dysconnectivity further demonstrate a synergistic effect on the delayed Benson Figure copy. Our work implies that both tau-PET and WM connectivity could be helpful imaging biomarkers for screening for AD-related dementia. To our knowledge, this is the first study that evaluates the combined effect of tau and WM dysconnectivity on neurocognition. Our study could encourage more future work on applying multi-modality/cross-modality neuroimaging to explore useful imaging biomarkers and better understand the specific neurodegeneration process that each memory task can infer.

## Supplementary Material

2. Supplementary Table and Figures

## Figures and Tables

**Table 1:** Study demographics

	CN (*n* = 57)	MCI (*n* = 26)	Effect size	*P* value	# Samples
Age (years)	69.4 (6.9)	71.5 (8.3)	−0.12	ns	57/26
Sex (male, female)	15:42	13:13	0.20	ns	57/26
Education (years)	16.7 (2.6)	16.2 (2.8)	0.09	ns	57/26
Aβ PET positive (no, yes)	43: 11	10: 12	0.29	< .01	54/22
APOE ε4 positive (no, yes)	27: 23	10: 14	0.08	ns	50/24
MoCA	0.2 (1.2)	−1.4 (1.3)	0.57	<0.001	56/25
Rey AVLT (lm.)	−0.3 (0.9)	−1.9 (1.2)	0.61	<0.001	50/24
Rey AVLT (Del.)	0 (0.9)	−1.8 (1.4)	0.67	<0.001	53/24
Craft Stories (lm.)	0 (1.2)	−1.4 (1.1)	0.56	<0.001	56/26
Craft Stories (Del.)	0.1 (1)	−1.6 (1)	0.78	<0.001	56/26
Benson Figure Recall (lm.)	0 (1.1)	−0.4 (1.6)	0.12	ns	56/26
Benson Figure Recall (Del.)	0.2 (0.9)	−2.3 (1.8)	0.73	<0.001	56/26
ns: not significant					
mean (std)					
lm.: immediate					
Del.: delayed					

## Data Availability

The datasets (but not the original images) and codes generated and/or analyzed during the current study are available from the corresponding author on reasonable request.
