# Supplementary material for: Tau deposition and structural connectivity demonstrate differential association patterns with neurocognitive tests
Source: Brain Imaging Behav. 2021 Sep 17;16(2):702–14. doi: 10.1007/s11682-021-00531-7 (PMC8935446; doi:10.1007/s11682-021-00531-7)

Supplementary Table S1: ROIs contain associations between global amyloid deposition (i.e., global cortex centiloid value) and connectivity metrics (*Spearman correlation, non-FRD, p<0.05*).

Supplementary Figure S1: Group mean connectivity matrix (i.e., streamline density) and Strength map extracted using different thresholding filtering (Strength map is normalized the ROI with the maximum value for ease of comparison between different filtering schemes).


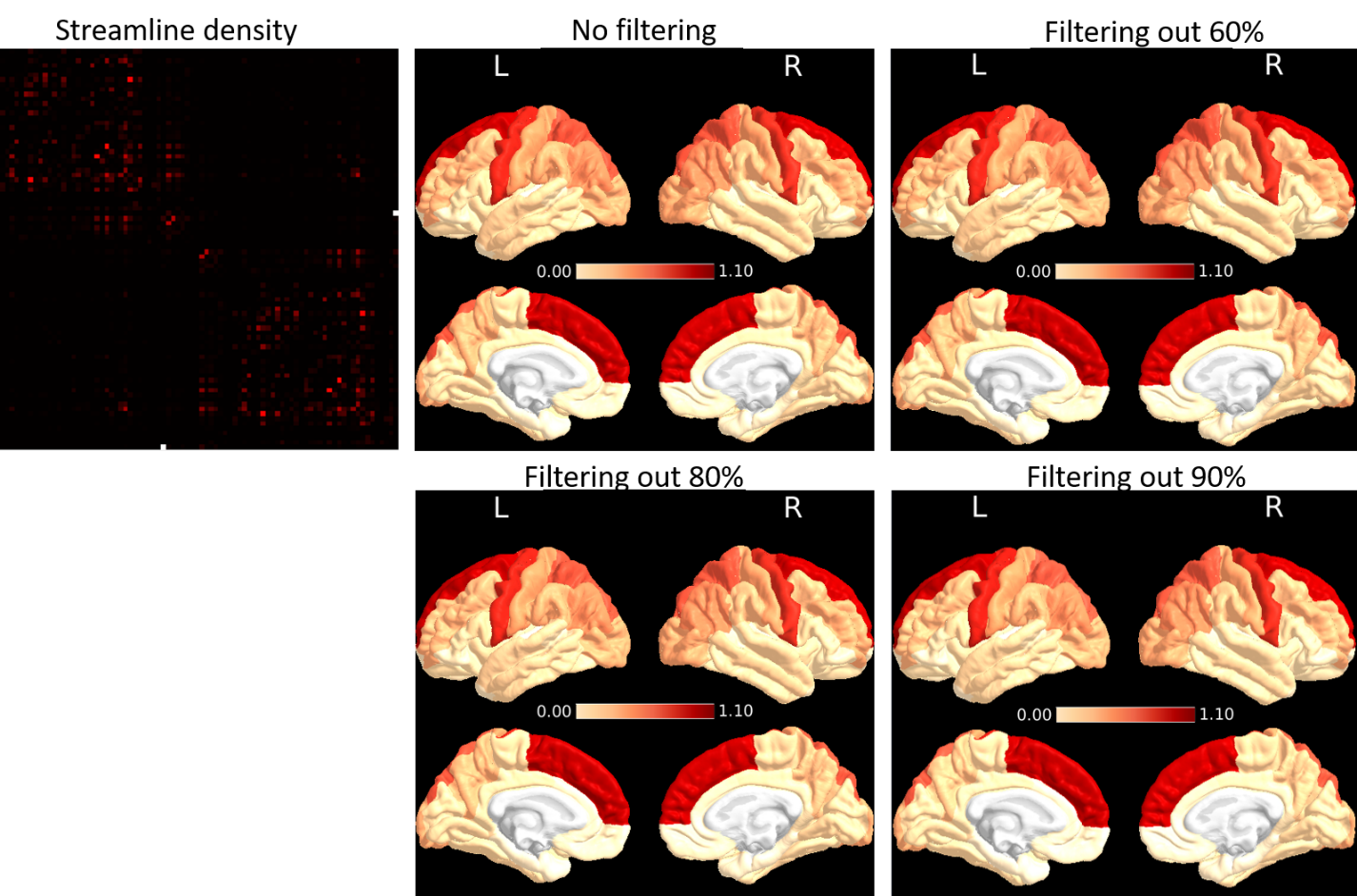


Supplementary Figure S2: Tau correlations with neurocognitive test results without FDR correction. Darker colors indicate stronger negative correlations, while lighter colors indicate weaker negative correlations. Regions with *P* > 0.05 appear white.

Abbreviations: L = Left, R = Right, MOCA = Montreal Cognitive Assessment, Rey AVLT = Rey Auditory Verbal Listening Test, Im. = Immediate, Del. = Delayed.


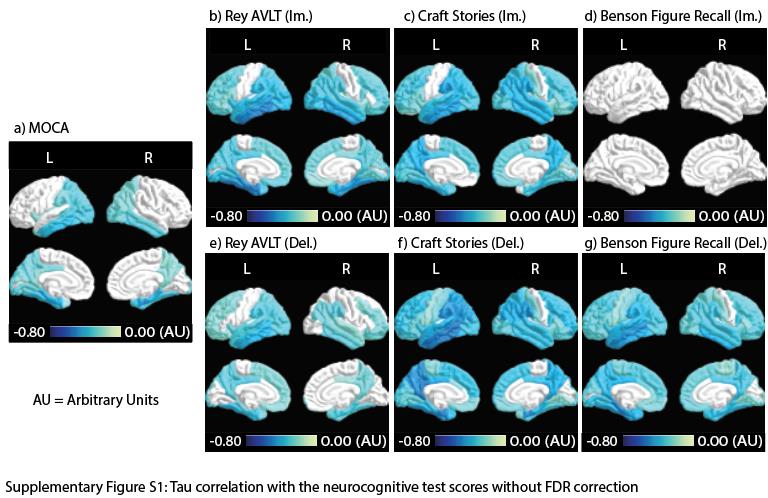


Supplementary Figure S3: Cluster coefficient correlation with neurocognitive test results without FDR correction. Blue regions indicate negative correlation, with darker colors signifying stronger correlations. Red/orange regions indicate positive correlations, with darker colors signifying stronger correlations. Regions with *P* > 0.05 appear white.

Abbreviations: L = Left, R = Right, MOCA = Montreal Cognitive Assessment, Rey AVLT = Rey Auditory Verbal Listening Test, Im. = Immediate, Del. = Delayed.


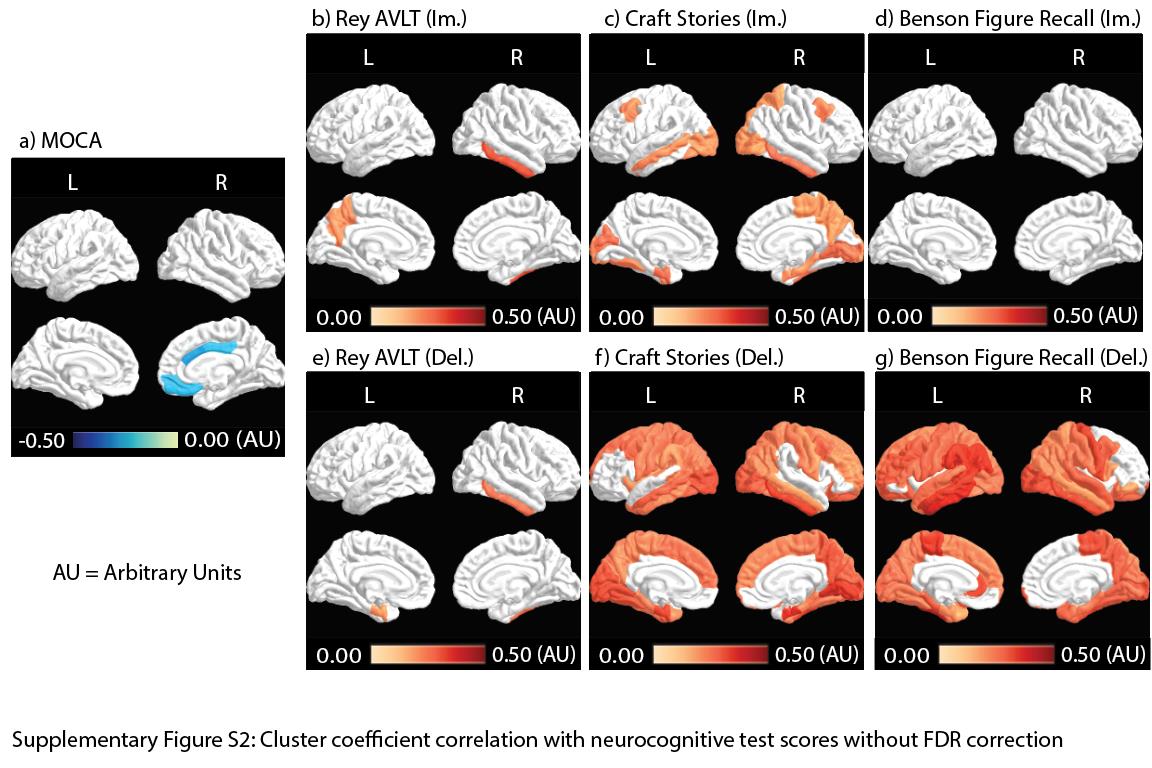


Supplementary Figure S4: Strength Correlations with neurocognitive test results with and without FDR correction (upper and lower panel respectively). Darker colors indicate stronger positive correlations, while lighter colors indicate weaker positive correlations. Regions with *P* > 0.05 appear white.

Abbreviations: L = Left, R = Right, MOCA = Montreal Cognitive Assessment, Rey AVLT = Rey Auditory Verbal Listening Test, Im. = Immediate, Del. = Delayed.


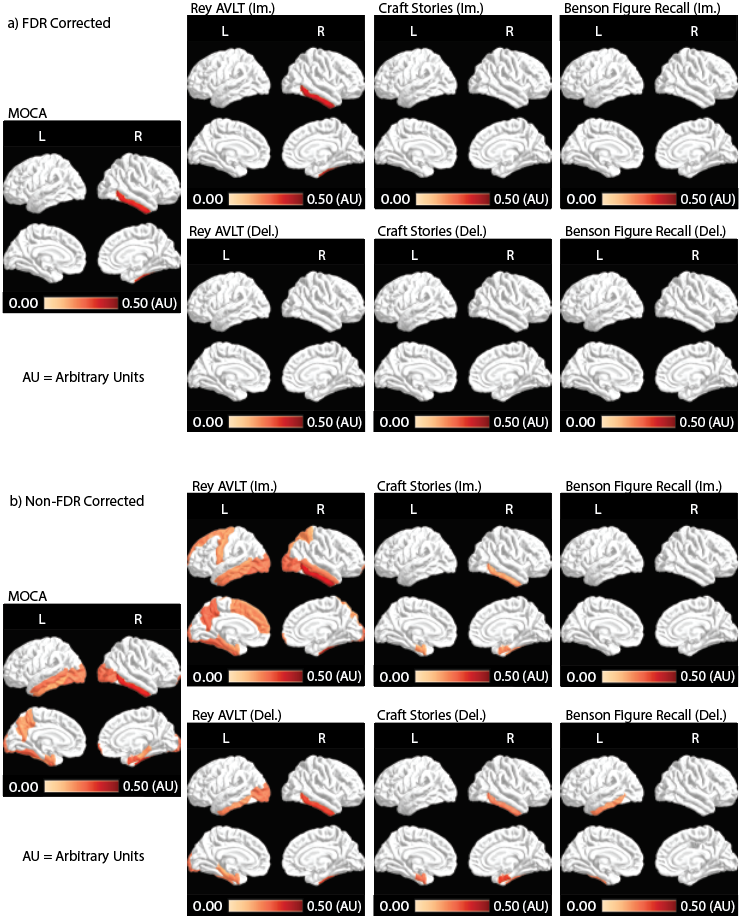


Supplementary Figure S5: Scatter plots of main Figures 2&3 with amyloid-β (Aβ) Status

**
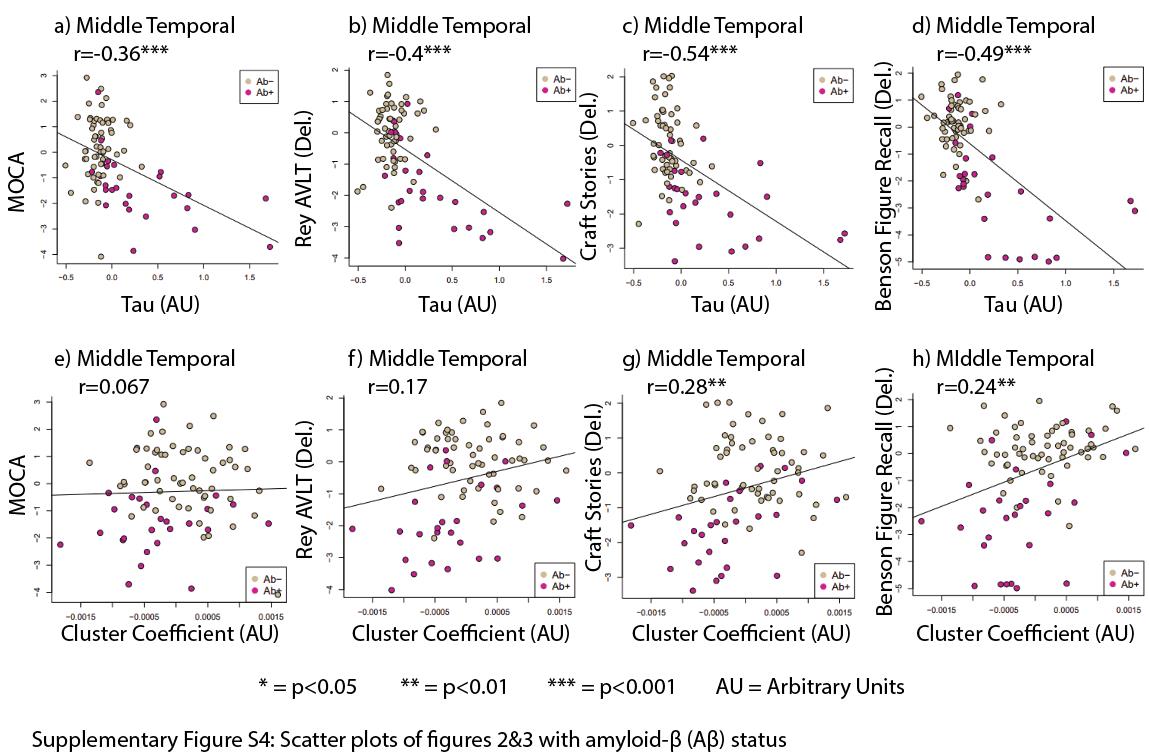
**

Supplementary Figure S6: Within group analysis for Aβ- and Aβ+. a). Tau correlations with memory tests. b). Cluster coefficient correlations with memory tests. Color-coded ROIs contain correlations with a significant level *P* < 0.05 (without FDR correction). Blue: negative correlation. Red: positive correlation. Regions with *P* > 0.05 appear white.

Abbreviations: L = Left, R = Right, MOCA = Montreal Cognitive Assessment, Rey AVLT = Rey Auditory Verbal Listening Test, Im. = Immediate, Del. = Delayed.


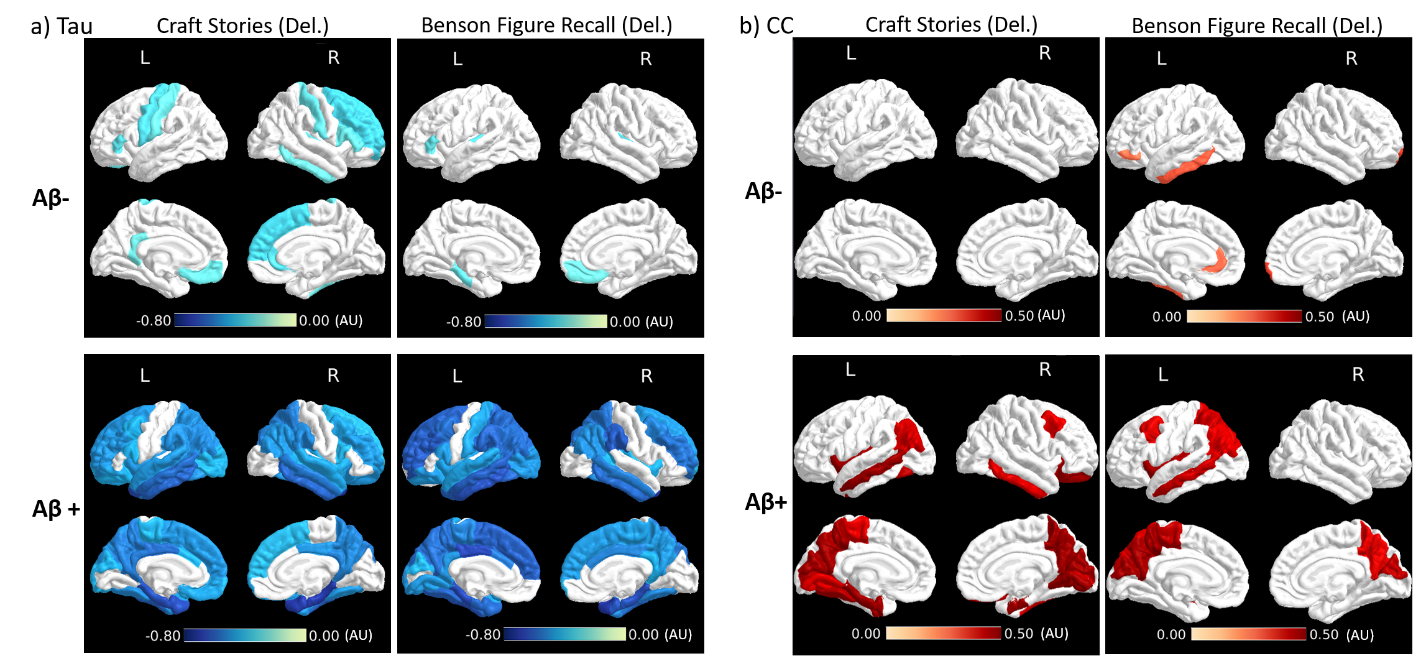


Supplementary Figure S7: Within group analysis for Aβ- and Aβ+. a). Tau correlations with memory tests. b). Cluster coefficient correlations with memory tests. Color-coded ROIs contain correlations with a significant level *P* < 0.05 (without FDR correction). Blue: negative correlation. Red: positive correlation. Regions with *P* > 0.05 appear white.

Abbreviations: L = Left, R = Right, MOCA = Montreal Cognitive Assessment, Rey AVLT = Rey Auditory Verbal Listening Test, Im. = Immediate, Del. = Delayed.


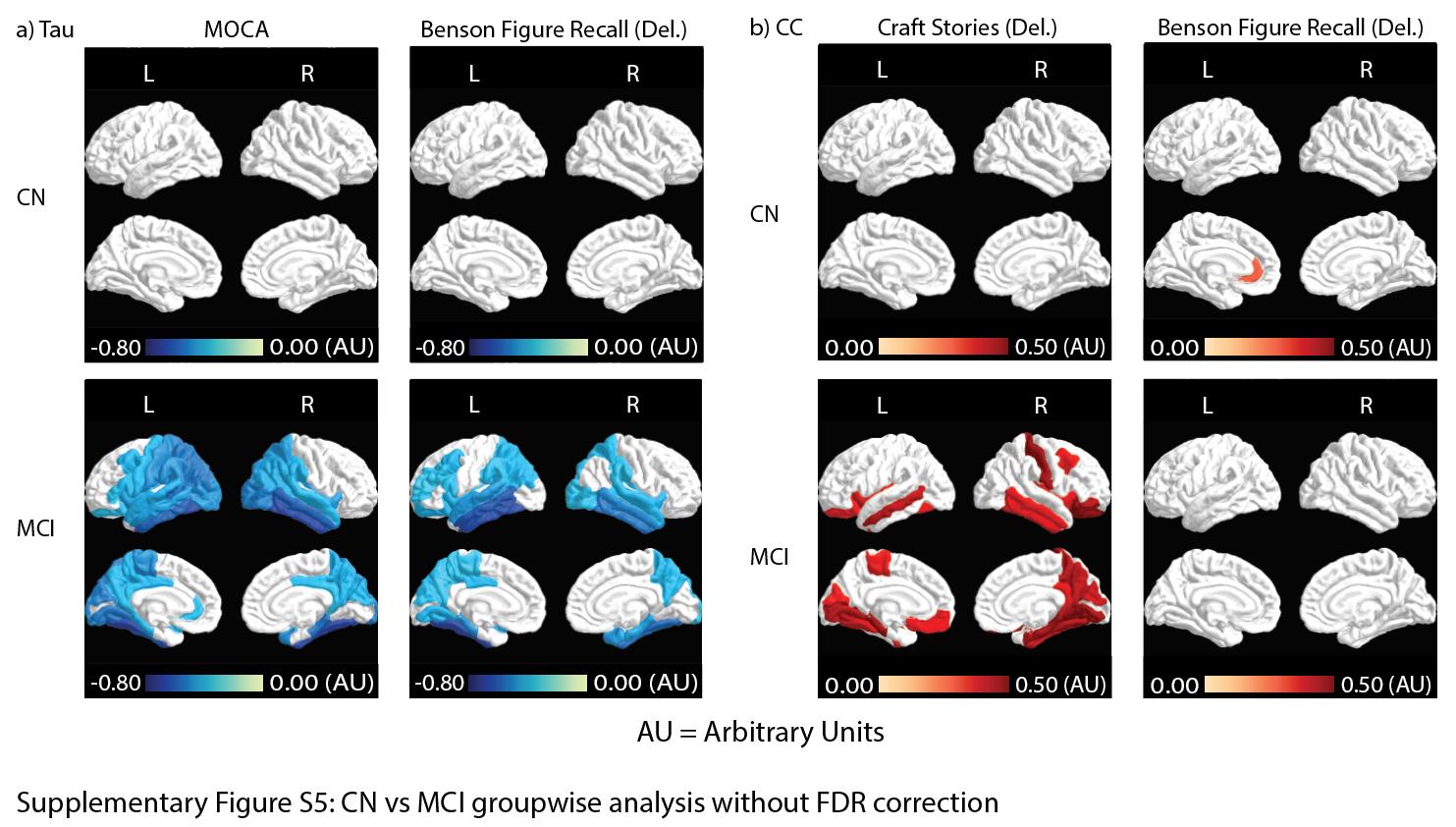

Supplement: Supplementary file 1 — (DOCX 2984 kb) [file 11682_2021_531_MOESM1_ESM.docx]
